# Effects of SNPs on TNF-α and IL-10 cytokine expression in TB and HIV patients in the Capricorn district, Limpopo Province, South Africa

**DOI:** 10.4314/ahs.v24i1.2

**Published:** 2024-03

**Authors:** Mosebo A Manabile, Tibello C Maguga-Phasha, Marema E Makgatho

**Affiliations:** Pathology and Medical Sciences, University of Limpopo, Limpopo Province, South Africa

**Keywords:** Tuberculosis, Human immunodeficiency virus, interleukin-10

## Abstract

**Background:**

The impact of Tuberculosis (TB) places an immense burden on the health care system. Infection with Human Immunodeficiency Virus (HIV) is a significant risk factor in the development and progression of TB disease. Single Nucleotide Polymorphisms (SNPs) in the promoter region of Interleukin-10 (IL-10) and Tumour Necrotic Factor-Alpha (TNF-α) may play a major role in the disease mechanism and understanding these mechanisms might prove to be a useful diagnostic tool in evaluating the immune regulation and progression of the disease.

**Objective:**

This study aimed to determine the relationship between cytokine levels and gene variants of Interleukin-10 and Tumour Necrotic Factor Alpha in TB and HIV-infected participants.

**Methods:**

Cytokine levels were determined by ELISA, and SNPs were determined by MassArray®.

**Results:**

The levels of TNF-α were higher in the TB group than the HIV (p < 0.001) and TB-HIV (p = 0.011) groups, but similar to the TNF-α levels in the control group. In the HIV group, IL-10 levels were higher than those of the TB (p < 0.001) and control groups (p = 0.039), whereas there was no difference between the IL-10 levels in the HIV and the TB-HIV infection groups. The ratio was determined and there were no differences between the four infection groups. In this study, no associations were detected between the circulating plasma levels of TNF-α and IL-10 and their genotypes.

**Conclusion:**

Our data showed that the gene variants were not associated with circulating plasma levels of TNF-α and IL-10 in our study population. A pro-inflammatory environment was found in the TB and TB-HIV groups, which is suggesting of bacterial clearance, while an anti-inflammatory environment was found in the HIV group, which suggests the suppression of viral replication.

## Introduction

Tuberculosis (TB), caused by *Mycobacterium tuberculosis*, remains a major global challenge in developing countries despite the introduction of national and international control strategies 1,2 According to the World Health Organization, an estimated 10.0 million new cases of TB occurred in 2017 worldwide. South Africa is one of only six countries which account for two-thirds of all active TB cases 3. Studies also indicate that TB-infected pregnant women may have a 32-fold increased risk of death if co-infected with HIV 4.

To reduce the burden of TB and the progression of HIV infections, it is necessary to identify individuals at high risk 5. The balance between Th1 and Th2 host immune responses plays a vital role in the pathogenesis of TB and HIV infections by interfering with naïve T-cell activation, which may result in a distorted immune response 6,7 TNF-α is a pleiotropic pro-inflammatory cytokine that is mainly produced by macrophages, monocytes, and dendritic cells (DC) 5. The main function of TNF-α cytokine is to induce apoptosis in cells infected with MTB, while it promotes viral replication in HIV infected cells 8-10. IL-10 is an immunoregulatory cytokine that plays an important role in suppressing/controlling the inflammatory response in both TB disease and HIV infection 11.

Single Nucleotide Polymorphisms (SNPs) have been identified in the promoter regions of TNF-α and IL-10, and they have been postulated to affect normal cytokine production 12. Several studies have investigated the roles of SNPs in the promoter regions of TNF-α and IL-10 in TB susceptibility and HIV progression, but findings remain inconclusive 12,11,13,5,14. Understanding the effects of SNPs on circulating plasma levels of cytokines may help identify individuals at high risk of susceptibility or progression of TB and HIV infections. This study aimed to investigate the relationship between cytokine production and their SNPs in the promoter regions of TNF-α (rs1800629 and rs361525) and IL-10 (rs1800896 and rs1800872) in TB-infected participants.

## Methods

### Study group

A case-control and experimental study to this effect was conducted in the Capricorn District Municipality of the Limpopo Province, South Africa. It was conducted between the years 2015–2017, using the primary health care clinics under the Polokwane/Mankweng Hospital complex. A questionnaire translated into the local language (Sepedi) was used to recruit volunteers. Assent was obtained from parents for study participants under 18. There were 116 participants who had either TB (39 participants), HIV (40 participants), or both (TB-HIV with 37 participants), and 40 participants who had neither of these conditions. The study was approved by the Turfloop Research Ethics Committee at the University of Limpopo, permission was obtained from the Department of Health, Office of the clinical manager, and approval from the Capricorn District Office for the use of clinics and hospitals. Informed consent was obtained from each participant.

### Sputum samples

Sputum samples were decontaminated using the N-acetyl-L-cysteine-sodium hydroxide (NALC-NaOH) method, cultured on Lowenstein-Jensen (LJ media (Merck, German), and sputum slide smears were stained using the Ziehl-Neelsen (ZN) staining method to confirm the presence of *Mycobacterium tuberculosis*.

### DNA extraction

A total of 5 ml of blood per participant was collected in an ethylenediaminetetraacetic acid (EDTA) tube and centrifuged at 3000 rpm for 10 minutes. The plasma was separated and stored at -80°C until analysis. Genomic DNA was extracted from whole blood (1–2 ml) using a commercially available DNA extraction kit (PureLink Genomic DNA Mini Kit) according to the manufacturer's guidelines. Genomic DNA quality and quantity was confirmed by NanoDrop™ 2000 (Thermo Fisher Scientific, USA) and gDNA was stored at -20°C until further use.

### Serum levels of TNF-α and IL-10

Plasma samples were used to determine the circulating plasma levels of TNF-α and IL-10 using a Quantikine ELISA kit (R&D System), and HIV status was determined using the Alere Determine™ HIV–1/2 Ag/Ab Combo commercial kit. The plasma, which had been stored at -80°C, was subjected to the assay method. All reagents were brought to room temperature, and the plasma samples were allowed to thaw once for about an hour before use. All the reagents, working standards and protocols were done according to the manufacturer's instructions. The absorbance was read using an ELISA plate reader (BIO-RAD) at 450 nm and 570 nm. The detection range of TNF-α was 15.6–1000 pg/ml, and that of IL-10 was 7.8–500 pg/ml. All the samples were assayed in duplicate. Single Nucleotide polymorphisms of TNF-α and IL-10 Extracted gDNA samples were genotyped for TNF-α (308 G/A; 238 G/A) and IL-10 (1082 T/C; 592 T/G) using MassArray® (MA) Designer Software (Agena Bioscience, USA). The designed primers (Table 1) were obtained commercially from Inqaba Biotech (Pretoria, South Africa). All the reagents (Master Mix) and protocol were used following the manufacturer's instructions.

**Table 1:** The forward and reverse primers for TNF-α and IL-10 used in the study

Primers		Forward Sequence	Reverse Sequence
**TNF-A**	308 G/A rs1800629	5′-GGAGGCAATAGGTTTTGAGG-3′	5′-GGGTCCTACACACAAATCAG-3′
	238 G/A rs361525	5′-CACACAAATCAGTCAGTGGC-3′	5′-GAGGGGTATCCTTGATGCTT-3′
**IL-10**	1082T/C rs1800896	5′-ATTCCATGGAGGCTGGATAG-3′	5′-GAAGCCTTAGTAGTGTTGTC-3′
	592 T/G rs1800872	5′-TCCTCAAAGTTCCCAAGCAG-3′	5′-GATGTGTTCCAGGCTCCTTT-3′

### Statistical analysis

Statistical analysis was performed using the Statistical Package for Social Science version 24 (SPSS v.24) software. Descriptive statistics were used to determine the demographics and the differences between infection groups were achieved using One-Way ANOVA. The Kruskal-Wallis, or one-way ANOVA, a non-parametric test, was used to compare cytokine levels by infection group. Bivariate and Pearson correlation analysis was used to correlate the gene loci and cytokine levels in different infection categories. All the results were adjusted by Bonferroni correction for multiple tests. The logistic regression model was used to obtain the Odds Ratio (OR) and 95% Coefficient Intervals (95% CI). A p-value of ≥ 0.05 was considered statistically significant.

## Results

This study included 156 participants, 104 of whom were female and 52 of whom were male. Participants who were infected with TB, HIV, or both were significantly younger than the participants in the control group. There was a significant association within the infection group with gender (p = 0.022), age (p = 0.001), and patients with at least one chronic infection (p = 0.002). The proportion of females among the TB-infected participants was significantly higher than the proportion of females in the TB-HIV-infected group, while the proportion of males among TB-infected participants was significantly lower than the proportion of males among the TB-HIV co-infected participants. The control group had the highest proportion of participants, with at least one chronic infection compared to the other three groups (Table 2).

**Table 2:** Characteristics of the study participants

Participant characteristics		TB (N=39)	HIV (N=40)	TB-HIV (N=37)	Controls (N=40)	P-value
**Age (mean ± SD)**		41.03 ± 15.28 a	41.75 ± 13.40 b	41.24 ± 12.07 c	56.13 ± 20.48 a,b,c	0.001
	Male	7 (17.9) *	13 (32.5)	19 (51.4) *	13 (32.5)	
**Gender**	N (%)				0.02
	Female	32 (82.1) *	27 (67.5)	18 (48.6) *	27 (67.5)	
	N (%)					
**At least 1-Chronic Infection n (%)**	11 (28.2)	5 (12.5) n	5 (13.5) m	18 (45.0) n,m	0.002

* p-value < 0.05 for TB versus TB-HIV, n p < 0.05 for HC versus HIV, m p < 0.05 for TB-HIV versus HC, TB-HIV a p-value < 0.05 for HC versus TB, b p-value < 0.05 for HC versus HIV, c p-value < 0.05 for HC versus TB-HIV

TB- Tuberculosis, HIV - Human Immunodeficiency Virus.

In this study, the participants infected with HIV were on.

### Circulating plasma levels (TNF-A And Il-10) and TNF-A/Il10 ratio

**Figure 1:**
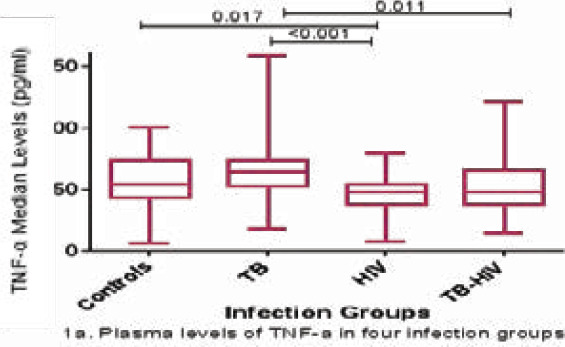

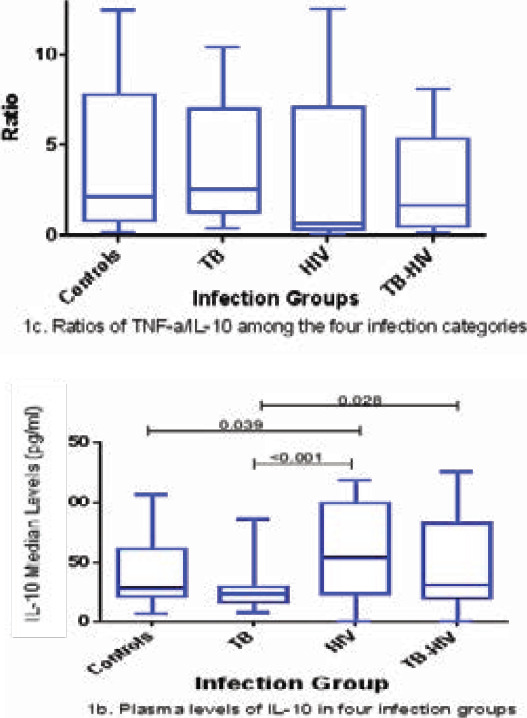
TNF-α and IL-10 plasma levels in various infection groups (1a, 1b and 1c). Data are presented as box plots. The box represents the first through to the third quartiles; middle line is the median, and the lines outside represent the minimum and maximum values (outliers excluded)

The levels of TNF-α were significantly higher in the TB-infected participants than in the HIV-infected (p < 0.001) and HIV-TB co-infected (p = 0.011) participants as shown in Figure 1a. The levels of TNF-α were significantly lower in the HIV-infected participants than in the control participants (p = 0.017). However, the levels of TNF-α between control participants and those infected with TB, between the control participants and the TB-HIV-infected group, and between the HIV and TB-HIV groups, were not significantly different (Figure 1a). HIV-infected participants had significantly higher levels of IL-10 than the TB-infected participants (p = 0.0001) and the control participants (p = 0.039). Participants with TB-HIV co-infection had significantly higher levels of IL-10 than those infected with TB only (p = 0.028). There was no significant difference in the IL-10 levels between control and TB-infected participants, control and co-infected participants, and HIV and co-infected participants (Figure 1b). Although there were no significant differences in the TNF-α/IL-10 ratio among the four categories, the highest ratio was observed in the TB group. HIV-infected participants tended to have an anti-inflammatory environment as shown by a ratio of less than 1, while TB and TB-HIV-infected participants depicted a pro-inflammatory environment, and control individuals also tended to have a pro-inflammatory environment (Figure 1c).

### Relationship between SNPS of TNF-A (308 G/A and 238 G/A) and Il-10 (1082 T/C and 592 T/G) with their plasma levels

**Figure 2:**
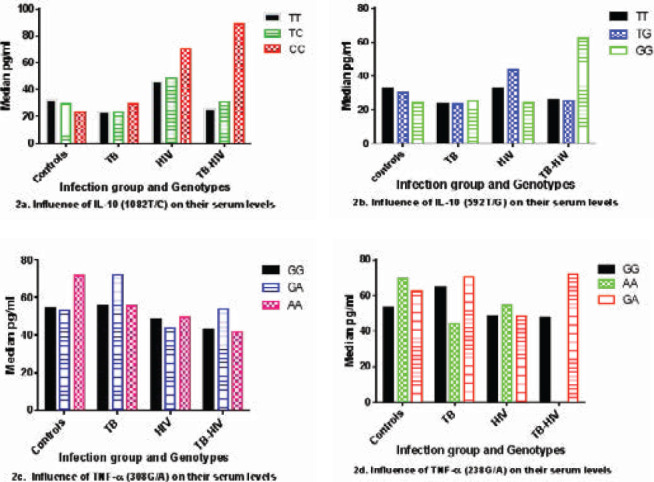
The influence of TNF-α and IL-10 genotypes on their circulating plasma levels in different infection groups (2a, 2b, 2c, and 2d)

There were no significant associations between the genotypes of TNF-α and IL-10 and their circulating plasma levels. Some genotypes such as the homozygous Wild-type of both IL-10 (1082 T/C and 592 T/G) in the HIV and TB-HIV group as shown in Figure 2a and the GA genotypes of TNF-α (308 G/A and 238 G/A) locus TB-HIV group in Figure 2b showed a slightly increased expression of the cytokines. The relationship between TNF-α cytokines and their SNPs is shown in Figures 2a, 2b, 2c, and 2d).

## Discussion and conclusion

This study evaluated the influence of SNPs on the circulating plasma cytokine levels of TNF-α and IL-10. To our knowledge, this study is the first to investigate the IL-10 (-1082 T/C and IL-10 -592 T/G) gene variants in TB, HIV and TB-HIV co-infected individuals. There were no statistical differences found across the infection groups. There have been a few studies hat have shown a correlation between circulating plasma levels and their SNPs. A study in India found that the AG genotype of TNF-α (308 A/G) and the AG genotype of IL-10 (1082 G/A) significantly correlated with TB disease 5. Geographic location and distinctly different genetic backgrounds may be contributing factors to our different results.

This study further evaluated the circulating plasma levels of IL-10 and TNF-α cytokines in TB, HIV, and TB-HIV infection groups. To our knowledge, this work is the first to report on the circulating plasma levels of TNF-α and IL-10 among patients infected with TB, HIV, and TB-HIV in Limpopo Province, South Africa. Although the study found higher levels of TNF-α in the TB-infected group than among the HC, levels of significant difference were not found. Several previous studies have found increased plasma levels of TNF-α in TB disease compared to control groups 4,12-14. It is important to note that participants in the control group in this study were much older than those in the TB group. The majority of participants in the control group had chronic conditions such as diabetes and hypertension, which are known to be prevalent in elderly people 15 and are commonly accompanied by elevated TNF-α levels 12,16.

Lower levels of TNF-α were found in the HIV-infected group compared to the control group 17, although there are studies that have reported increased levels in HIV compared to the control group 14. If one puts aside the influence of the other chronic infections on the expression of TNF-α, studies have reported that the expression of TNF-α levels in HIV infected patients in the acute phase of HIV does exhibit elevated levels of pro-inflammatory cytokine profiles such as INF-γ, TNF-α, IL-2, and IL-12, which will steadily subside as the disease progresses to a chronic phase 18,19. This might be the reason behind the decreased levels in the HIV infected participants in this study and these decreased levels of TNF-α in immunocompromised patients may increase the risk of secondary infections such as TB.

The circulating plasma levels of TNF-α cytokine were found to be significantly lower in the TB-HIV group compared to the TB group, as has been reported in other studies 4,20. The decreased levels of TNF-α cytokine in the TB-HIV group may be due to a more pronounced reduction of CD4+, CD8+ and macrophages, which will in turn decrease the production of TNF-α cytokine. A correlation between reduced pro-inflammatory cytokines with reduced T-cell function has been reported previously 21, which might lead to the progression of both TB and HIV infections. There was no difference in the plasma levels of TNF-α cytokine between the TB-HIV group and HIV groups in this study. Studies have noted the influence of ART usage in reducing the expression of pro-inflammatory cytokines in HIV-infected patients, and it has also been reported that the influence of ART dominates in dually infected participants 4,14,22 This may possibly be the reason why there were no differences in the TNF-α expression between the HIV and TB-HIV infection groups in this study.

Several studies have evaluated the expression of IL-10 cytokine levels in TB-infected participants, and found similar plasma levels of IL-10 in the participants with active TB and the control group, as was the case in this study 5,6,8. Other studies have reported high levels of IL-0 before the start of TB treatment when compared to the control group, but also reported a gradual decrease of IL-10 levels during the course of TB disease 6,23 These findings suggest the effect of TB treatment on the circulating plasma IL-10 levels. These results also emphasize the crucial role of IL-10 in the pathogenesis of TB disease, where people with high levels of IL-10 at the end of treatment are most likely to fail to control the infection and may be prone to TB reactivation. This phenomenon in HIV infected participants where IL-10 levels were found to be high is supported by several studies 14,22,24,25. The balance between pro- and anti-inflammatory cytokines is crucial in the clinical outcome of TB and HIV infections 13. This study further investigated the micro-immune response of TNF-α/IL-10 ratios in TB, HIV, and TB-HIV infection groups, in comparison with a control group. Discounting the non-significant findings in the study, a high ratio of IL-10 in the HC, TB, and TB-HIV infection groups was found, a factor that is supported by another research 5,26. The increased ratio of TNF-α/IL-10 in the HC group further suggests the involvement of other chronic infections 22, whilst the increased ratio of TNF-α/IL-10 in TB and TB-HIV participants likely marks a stage in the illness where the immunologic response is attempting to contain or clear the bacilli 2.

The lower TNF-α/IL-10 ratio in HIV-infected participants shows an anti-inflammatory environment characterized by a shift of Th1 response to Th2 response. Previous studies have reported a negative association of IL-10 cytokine levels with CD4+ reduction, and an imbalance of Th1/Th2 cytokines with a Th2 micro-environment in later stages of HIV infection 5,14,24,25,26,28. The results of this work demonstrate the correlation of circulating plasma levels with different infection groups. The cytokines, together with their ratios (TNF-α/Il-10), can be used as indicators or biomarkers in different groups. The lack of association between the plasma levels and the SNPs suggests that such studies should be done between populations where genetic background differs significantly, not at the provincial level, and most probably between countries. It is also possible that infecting strain differences could play a role in cytokine responses. There are other associations that could occur between gene variants (HMGB1, IL-4, IL-12, TGF-beta) and the diseases in question. Cytokines have the potential to be used as biomarkers for Infection Risk, Progression, and Outcome. Such studies should be prioritized in future to assist in developing new diagnostic techniques. Other factors that need to be studied further include the variability of receptors of the cytokines, the expression of different pathways such as cGAS-STING pathway and factors that control it, and the variations in the levels of various cytokines during different stages of infection between different populations and dietary habits of the participants.
